# Photobiomodulation preserves behaviour and midbrain dopaminergic cells from MPTP toxicity: evidence from two mouse strains

**DOI:** 10.1186/1471-2202-14-40

**Published:** 2013-03-27

**Authors:** Cécile Moro, Napoleon Torres, Nabil El Massri, David Ratel, Daniel M Johnstone, Jonathan Stone, John Mitrofanis, Alim-Louis Benabid

**Affiliations:** 1 CEA, LETI, CLINATEC, Grenoble, 38054, France; 2 Department of Anatomy & Histology, University of Sydney, Sydney, Australia; 3 Department of Physiology, University of Sydney, Sydney, Australia

**Keywords:** Tyrosine hydroxylase, Substantia nigra, Balb/c, C57BL/6, Neuroprotection

## Abstract

**Background:**

We have shown previously that near-infrared light (NIr) treatment or photobiomodulation neuroprotects dopaminergic cells in substantia nigra pars compacta (SNc) from degeneration induced by 1-methyl-4-phenyl-1,2,3,6-tetrahydropyridine (MPTP) in Balb/c albino mice, a well-known model for Parkinson’s disease. The present study explores whether NIr treatment offers neuroprotection to these cells in C57BL/6 pigmented mice. In addition, we examine whether NIr influences behavioural activity in both strains after MPTP treatment. We tested for various locomotive parameters in an open-field test, namely velocity, high mobility and immobility.

**Results:**

Balb/c (albino) and C57BL/6 (pigmented) mice received injections of MPTP (total of 50 mg/kg) or saline and NIr treatments (or not) over 48 hours. After each injection and/or NIr treatment, the locomotor activity of the mice was tested. After six days survival, brains were processed for TH (tyrosine hydroxylase) immunochemistry and the number of TH^+^ cells in the substantia nigra pars compacta (SNc) was estimated using stereology. Results showed higher numbers of TH^+^ cells in the MPTP-NIr groups of both strains, compared to the MPTP groups, with the protection greater in the Balb/c mice (30% vs 20%). The behavioural tests revealed strain differences also. For Balb/c mice, the MPTP-NIr group showed greater preservation of locomotor activity than the MPTP group. Behavioural preservation was less evident in the C57BL/6 strain however, with little effect of NIr being recorded in the MPTP-treated cases of this strain. Finally, there were differences between the two strains in terms of NIr penetration across the skin and fur. Our measurements indicated that NIr penetration was considerably less in the pigmented C57BL/6, compared to the albino Balb/c mice.

**Conclusions:**

In summary, our results revealed the neuroprotective benefits of NIr treatment after parkinsonian insult at both cellular and behavioural levels and suggest that Balb/c strain, due to greater penetration of NIr through skin and fur, provides a clearer model of protection than the C57BL/6 strain.

## Background

Parkinson’s disease is a major movement disorder characterised by the distinct signs of resting tremor, akinesia and/or lead pipe rigidity [1,2]. These arise after a substantial loss of dopaminergic cells, mainly within the substantia nigra pars compacta (SNc) of the midbrain [3,4]. The factors that generate this cell loss are not entirely clear, but there is evidence for mitochondrial dysfunction as a result of exposure to an environmental toxin (eg MPTP (1-methyl-4-phenyl-1,2,3,6-tetrahydropyridine)) [5] and/or the presence of a defective gene [6].

Many previous studies have shown that some substances, such as anti-oxidants like CoQ10 (coenzyme Q10) [7] and melatonin [8], help neuroprotect dopaminergic cells in the SNc against degeneration in animal models of Parkinson’s disease. These substances are thought to reduce mitochondrial dysfunction by lessening the oxidative stress caused by free radicals generated by defective mitochondria present in Parkinson’s disease. In addition to these substances, recent studies have reported on the neuroprotective properties of low intensity light therapy, known also as photobiomodulation or near infra-red light (NIr) treatment, after parkinsonian insult. For example, NIr treatment protects neural cells *in vitro* against parkinsonian toxins such as MPTP and rotenone [9,10]. Further, we have shown that NIr treatment offers *in vivo* protection for dopaminergic cells in the SNc in an acute [11] and chronic [12] MPTP mouse (Balb/c) model. There is also a brief report indicating that NIr treatment improves the locomotor activity of mice after MPTP insult [13]. Although the mechanism of neuroprotection by NIr is not entirely clear, work on other systems indicate that NIr improves mitochondrial function and ATP synthesis in the damaged cells by increasing electron transfer in the respiratory chain and activating photoacceptors, such as cytochrome oxidase, within the mitochondria. Further, NIr has been shown to reduce the production of reactive oxygen species that are harmful to cells [14,15].

In this study, we sought to extend our earlier anatomical [11,12] and functional [16] studies by exploring the changes in locomotive behaviour of MPTP-treated mice after NIr treatment. Hitherto, this feature has not been reported extensively [13]. We undertook this behavioural analysis, together with a stereological account of SNc cell number, in two strains of mice, Balb/c (albino) and C57BL/6 (pigmented). This was done because there are reports that MPTP has differential effects on behaviour and dopamine levels in the basal ganglia in different strains of mice [17,18], as well as rats [19]. We wanted to determine whether there were mouse strain differences in the effect of NIr treatment after MPTP insult.

## Methods

### Subjects

Male BALB/c (albino; n=40) and C57BL/6 mice (pigmented; n=40) mice were housed on a 12 hr light/dark cycle with unlimited access to food and water. Animals were 8–10 weeks old. All experiments were approved by the Animal Ethics Committee of the University of Sydney and COMETH (Grenoble).

### Experimental design

**Figure 1:**
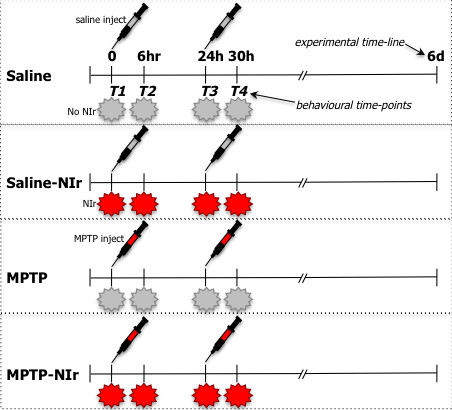
**Outline of the different experimental groups used in this study, namely Saline, Saline-NIr, MPTP, MPTP-NIr.** The experimental time-line and behaviour time-points are shown. For the experimental time-line, there were two injections (saline or MPTP) and they occurred in the first 24 hrs. There were four NIr treatments (or not) and these occurred immediately after each injection and about 6 hrs later on the same. After the last NIr (and fourth) treatment, mice were allowed to survive for 6 days thereafter. There were four behavioural time-points; (T1) after first injection and NIr (or no) treatment; (T2) after second NIr (or no) treatment; (T3) after second MPTP or saline injection and third NIr (or no) treatment; (T4) after fourth NIr (or no) treatment.

We set up four experimental groups (see Figure 1). Mice received intraperitoneal injections of either MPTP or saline, combined with simultaneous NIr treatments or not. The different groups were; (1) Saline: saline injections with no NIr (2) Saline-NIr: saline injections with NIr (3) MPTP: MPTP injections with no NIr (4) MPTP-NIr: MPTP injections with NIr. Each experimental group comprised ten mice of each strain.

Following our previous work, we used an acute MPTP mouse model [11,16]. The acute model is a well-accepted model of the disease [20,21] and has revealed many aspects of the mechanisms of Parkinson’s disease over the years. Although it does not provide information on the chronic progressive nature of the disease, it does generate mitochondrial dysfunction, dopaminergic cell death and a reduction in locomotive activity [20,21]. The latter two issues were central in this study, making the acute model most appropriate for our use. Briefly, we made two MPTP (25 mg/kg injections; total of 50 mg/kg per mouse) or saline injections over a 24 hour period. Following each injection, mice in the MPTP-NIr and Saline-NIr groups were treated to one cycle of NIr (670 nm) of 90 seconds from a light-emitting device (LED; Quantum Devices WARP 10). This treatment equated to ~0.5 Joule/cm^2^ to the brain [11]. Approximately 6 hours after each injection and first NIr treatment, mice in these groups received a second NIr treatment, but no MPTP or saline injection. Hence, each mouse in these groups received four NIr treatments, equalling ~2 joules/cm^2^ reaching the brain. This NIr treatment regime was similar to that used by previous studies, in particular, those reporting changes after trans-cranial irradiation [11,12,14-16]. For each treatment, the mouse was restrained by hand and the LED was held 1–2 cm above the head [11,12,16]. The LED generated no heat and reliable delivery of the radiation was achieved. For the Saline and MPTP groups, mice were held under the LED as described above, but the device was not turned on. After the last treatment, mice were allowed to survive for six days (Figure 1). This MPTP/NIr dose regime and survival period has been shown to furnish TH^+^ cell loss by MPTP and neuroprotection by NIr [8,11,16]. We also made some measurements of NIr penetration across the skin and fur of the two mouse strains. Skin was excised from the back of each mouse and positioned over a foil-coated vessel, with a calibrated light sensor at the bottom. NIr from the WARP-LED was then shone onto the skin and the penetration was recorded by the sensor (distance from WARP-LED to skin was ~4 cm and distance from skin to sensor was ~3 cm). For each strain, we compared the NIr penetration in cases where the fur was shaved from the skin to those that were unshaved. Each of the values obtained were compared to (and expressed as a percentage of) the values we recorded of NIr through the air, with no intervening skin.

Our experimental paradigm of simultaneous administration of parkinsonian insult and therapeutic application was similar to that of previous studies on animal models of Parkinson’s disease [8,11,12,16,22-24]. This paradigm is unlike the clinical reality where there is cell loss prior to therapeutic intervention. However, in our experimental study we hoped to determine the maximum effect of NIr neuroprotection.

### Immunocytochemistry and cell analysis

Following the survival period, mice were anaesthetised with an intraperitoneal injection of chloral hydrate (4%; 1 ml/100 g). They were then perfused transcardially with 4% buffered paraformaldehyde. The brains were removed and post-fixed overnight in the same solution. Next, brains were placed in phosphate-buffered saline (PBS) with the addition of 30% sucrose until the block sank. The midbrain was then sectioned coronally and serially (at 50 μm) using a freezing microtome. All sections were collected in PBS and then immersed in a solution of 1% Triton (Sigma) and 10% normal goat serum (Sigma) at room temperature for ~1 hour. Sections were then incubated in anti-tyrosine hydroxylase (Sigma; 1:1000) for 48 hours (at 4°C), followed by biotinylated anti-rabbit IgG (Bioscientific; 1:200) for three hours (at room temperature) and then streptavidin-peroxidase complex (Bioscientific; 1:200) for two hours (at room temperature). To visualise the bound antibody, sections were reacted in a 3,3^′^- diaminobenzidine tetrahydrochloride (Sigma) - PBS solution. Sections were mounted onto gelatinised slides, air dried overnight, dehydrated in ascending alcohols, cleared in Histoclear and coverslipped using DPX. Most of our immunostained sections were counterstained lightly with neutral red as well. In order to test the specificity of the primary antibody, some sections were processed as described above, except that there was no primary antibody used. These control sections were immunonegative.

In this study, we used TH immunocytochemistry to describe patterns of cell death and protection. As with many previous studies, we interpreted a change in TH^+^ cell number after experimental manipulation as an index of cell survival [8,11,12,22,23,25]. If cells lose TH expression, then they are likely to undergo death subsequently [25], which then leads to a reduction in Nissl-stained (and TH^+^) cell number [8,23]. Notwithstanding a small number of cells that may have transient loss of TH expression [26], a key aspect of our study was whether NIr treatment saved TH expression during a period when MPTP treatment alone would have abolished it [11,12]. In terms of analysis, the number of TH^+^ cells within the SNc was estimated using the optical fractionator method (StereoInvestigator, MBF Science), as outlined previously [8,11,12,23]. Briefly, systematic random sampling of sites - with an unbiased counting frame (100×100 μm) - within defined boundaries of SNc was undertaken. Counts were made from every second section, and for consistency, the right hand side of the brain was counted in all cases. All cells (nucleated only) that came into focus within the frame were counted and at least five sites were sampled per section.

Digital images were constructed using Adobe Photoshop (brightness and contrast levels were adjusted on individual images in order to achieve consistency (eg, illumination) across the entire plate) and Microsoft PowerPoint programmes.

### Behavioural analysis

During the experimental period, we performed a standard open-field test [17]. Mice were placed in white boxes (~20×20×20 cm) for C57BL/6 mice and black boxes for the Balb/c mice (this was important for software detection of contrast changes). Behavioural activity was measured and videotaped using a high definition camera (25000 images/sec) that detected changes in contrast and hence movement of mice. Mice were not acclimatised to the boxes prior to testing and boxes were cleaned thoroughly to avoid olfactory clues. Animal detection was made comparing a reference image that contained no subject with the live image containing the subject; the differences between the two were identified as subject pixel. Subject pixels changes were computed (Noldus, Ethovision, XT 8.5 version) to obtain different parameters of locomotor activity, for example velocity and mobility. Velocity was the mean speed of the mouse during trials (cm/sec) measured from the centre of gravity of the animal. To avoid “jittering”, a threshold of minimal distance moved of 0.3 cm was established. Mobility calculates the duration (in sec) during which the complete area detected as animal is changing even if the centre of gravity remains the same. High mobility refers to 10% or more of changes in percentage of body area detected between two samples, and immobility refers to less than 2% of changes. Each animal was tested at four time points (Figure 1); (T1) after first MPTP or saline injection and NIr (or no) treatment; (T2) after second NIr (or no) treatment; (T3) after second MPTP or saline injection and third NIr (or no) treatment; (T4) after fourth NIr (or no) treatment. Mice were tested for ~20 minutes at each time point. We tested locomotive activity at these points, particularly T1 and T3, because we wanted to explore the effects of NIr during a time when the MPTP was most effective (eg, immediately after injections), when the mice were most immobile and “sick” [17].

For comparisons between groups in the cell analysis, a one-way ANOVA test was performed, in conjunction with a Tukey-Kramer post-hoc multiple comparison test. For the behavioural analysis, groups were compared for time (T1,T2,T3,T4), drug (MPTP or not) and light (NIr or not) conditions using a three-way ANOVA test with a Bonferroni post-hoc test (using GraphPad Prism programme).

## Results

The results that follow will consider the cell and behavioural analyses for each strain separately.

### Cell analysis

**Figure 2:**
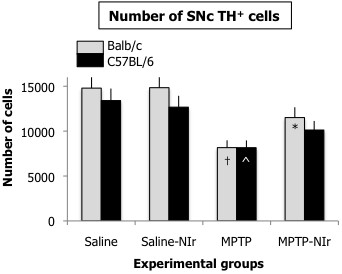
**Graph showing TH**^**+ **^** cell number in the SNc in the four experimental groups, in either the Balb/c (grey columns) or C57BL/6 (black columns) mice.** Columns show the mean ± standard error of the total number (of one side) in each group. There were ten animals per group. The symbols in the MPTP groups represent levels of significant difference in number from the Saline groups in each series, while symbols in the MPTP-NIr groups represent those from the MPTP groups; † represents p<0.001, ^ represents p<0.01 and * represents p<0.05.

Figure 2 shows the estimated number of TH^+^ cells in the SNc of the four groups in the Balb/c and C57BL/6 mice. Overall, the variations in number were significant for both Balb/c (ANOVA: F=4.9; p<0.001) and C57BL/6 (ANOVA: F=3.8; p<0.01) mice. For the Saline and Saline-NIr groups of both strains, the number of TH^+^ cells was similar; no significant differences were evident between these groups (Tukey test: p>0.05). For the MPTP groups, TH^+^ cell number was reduced compared to the saline control groups in both strains (~30%). These reductions were significant (Tukey test: p<0.05). In the MPTP-NIr groups, TH^+^ cell number was higher than in the MPTP groups of both strains, but more so in the Balb/c (~30%) compared to the C57BL/6 (~20%) mice. This increase reached statistical significance for the Balb/c group (Tukey test: p<0.05) but not the C57BL/6 group. Unlike the MPTP groups, the number of TH^+^ cells in the MPTP-NIr groups of both strains was not significantly different to the saline groups (Tukey test: p>0.05).

**Figure 3:**
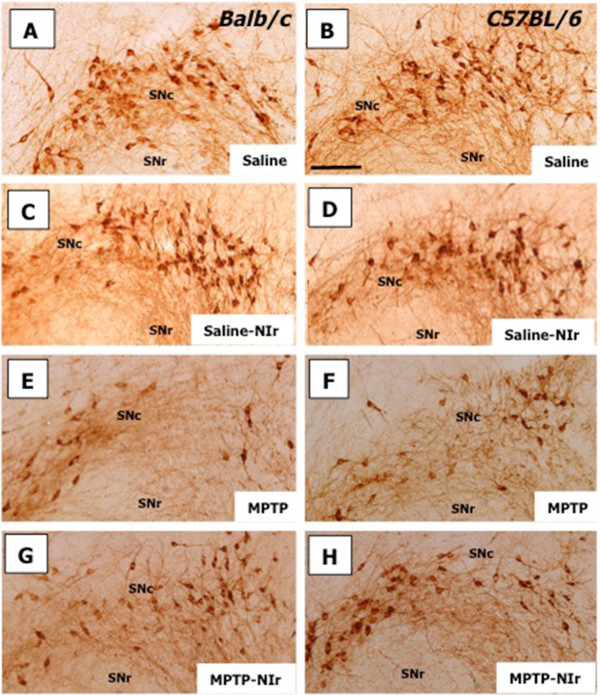
**Photomicrographs of TH**^**+ **^** cells in the SNc of Balb/c (A,C,E,G) and C57BL/6 (B,D,F,H) in each of the Saline (A,B), Saline-NIr (C,D), MPTP (E,F) and MPTP-NIr (G,H) groups.** Similar patterns of immunostaining were seen in both strains. There were fewer TH^+^ somata in the MPTP group (**E**,**F**) compared to other groups. All figures are of coronal sections; dorsal to top and lateral to right. The region depicted shows the lateral region of the SNc, corresponding approximately to plate 57 in the mouse atlas [27]. Scale bar = 100 μm.

These patterns are illustrated further in Figure 3 for both Balb/c (Figure 3A,C,E,G) and C57BL/6 (Figure 3B,D,F,H) in each of the Saline (Figure 3A,B), Saline-NIr (Figure 3C,D), MPTP (Figure 3E,F) and MPTP-NIr (Figure 3G,H) groups. Similar patterns of immunostaining were seen in both strains. Although there were fewer TH^+^ somata in the MPTP group (Figure 3E,F), those remaining were similar in overall appearance to those seen in the Saline (Figure 3A,B), Saline-NIr (Figure 3C,D) and MPTP-NIr (Figure 3G,H) groups. They had round or oval-shaped somata with one to two labelled dendrites.

### Behavioural analysis

**Figure 4:**
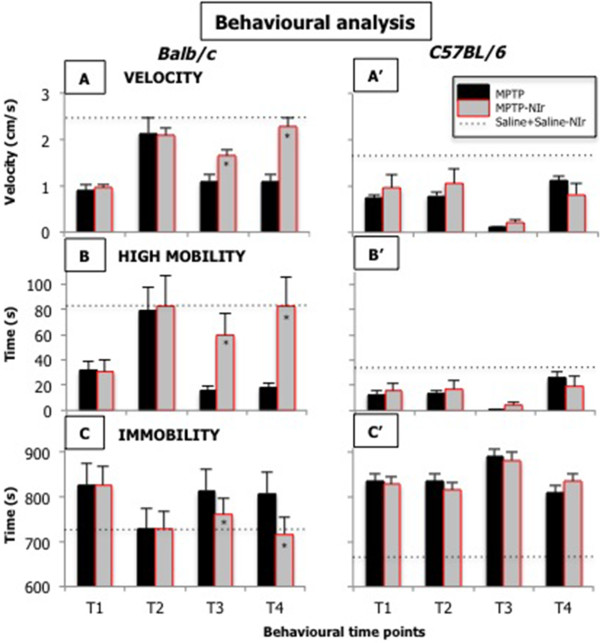
**Graphs showing the results of behavioural analysis of Balb/c (A,B,C) or C57BL/6 (A’,B’,C’) mice.** The _behavioural_ analysis included the locomotor activities of velocity (**A**,**A’**), high mobility (**B**,**B’**) and immobility (**C**,**C’**). Columns show the mean ± standard error of each group; black columns show results for MPTP groups, while grey columns show results for MPTP-NIr groups. There were ten animals per group. The asterisks (*) within the MPTP-NIr columns (**A**,**B**,**C**) represent p<0.05 level of significant difference in number from the MPTP group. The locomotor activity in the Saline and Saline-NIr groups were very similar in both strains; their values were pooled and represented as a dotted line across each of the graphs. Each animal was tested at four time points; (T1) after first MPTP or saline injection and NIr (or no) treatment; (T2) after second NIr (or no) treatment; (T3) after second MPTP or saline injection and third NIr (or no) treatment; (T4) after fourth NIr (or no) treatment.

Figure 4 shows recorded values of locomotor activity in Balb/c (Figure 4A,B,C) and C57BL/6 (Figure 4A’,B’,C’) mice, in terms of velocity (Figure 4A,A’), high mobility (Figure 4B,B’) and immobility (Figure 4C,C’). Overall, there were significant interactions for time and drug conditions for velocity, high mobility and immobility in both Balb/c (ANOVA: F range=7.5-13.6; p<0.05) and C57BL/6 (ANOVA: F range=16.8-40.5; p<0.05) mice, while significant interactions for time, drug and light conditions were evident for these locomotive activities in Balb/c (ANOVA: F range=11.7-24.2; p<0.05), but not in C57BL/6 (ANOVA: F range=0.4-0.8; p>0.05) mice.

The patterns of locomotor activity in the Saline and Saline-NIr groups were similar in both strains of mice. There was no significant effect of the light in the different time conditions (T1-T4) in the saline-treated cases (Bonferroni test: p>0.05). Hence, for clarity, the values of these groups were pooled and are represented as a dotted line across each of the graphs. By contrast, distinct changes in locomotor activity were evident between the MPTP and MPTP-NIr groups; their values are hence represented as individual columns at each time point (Figure 4). The results for each locomotor activity in the two strains will be considered separately below.

For Balb/c mice, at T1 (after first MPTP injection and NIr treatment) and T2 (after second NIr treatment) the locomotor activities in the MPTP and MPTP-NIr groups were similar. There were no significant effects of the light in these two time conditions in the MPTP-treated cases (Bonferroni test: p>0.05; Figure 4A,B,C). The effects of MPTP were immediate; compared to the saline control groups, both groups showed less velocity (Figure 4A) and high mobility (Figure 4B) and greater immobility (Figure 4C) at T1. By T2, there was considerable recovery of each locomotor activity in both MPTP and MPTP-NIr groups, with their values returning to control levels (Figure 4A,B,C). At T3 (after second MPTP injection and third NIr treatment) and T4 (after fourth NIr treatment), unlike at T1 and T2, there were significant effects of the light in the MPTP-treated cases (Bonferroni test: p<0.05; Figure 4A,B,C). At T3 and T4, the MPTP-NIr group had greater velocity (Figure 4A) and high mobility (Figure 4B) and less immobility (Figure 4C) than the MPTP group. Compared to the saline control groups, the MPTP-NIr group had similar locomotor activities at T3 and in particular, at T4 (Figure 4A,B,C). By contrast, the MPTP group at both T3 and T4, still had considerably less velocity (Figure 4A) and high mobility (Figure 4B) and greater immobility (Figure 4C) than the saline controls.

For C57BL/6 mice, there were distinct differences in locomotor activity compared to Balb/c mice. First, in C57BL/6 mice, there were no significant effects of the light at all time conditions (T1-T4) in the MPTP-treated cases (Bonferroni test: p>0.05; Figure 4A’,B’,C’); for Balb/c mice, there was no effect of the light in the MPTP-treated cases at T1 and T2 only (Figure 4A,B,C). Second, the MPTP and MPTP-NIr groups had considerably less velocity (Figure 4A’) and high mobility (Figure 4B’) and greater immobility (Figure 4C’) than the saline controls at the majority of the time points. In contrast to Balb/c mice, there was no evidence of NIr-specific recovery of function at T3 and T4; instead MPTP-treated mice appeared to have some recovery after the second MPTP injection (T4; Figure 4A’,B’,C’) irrespective of whether or not they received NIr treatment. Finally, control C57BL/6 mice showed lower baseline velocity (Figure 4A’) and high mobility (Figure 4B’), but also less immobility (Figure 4C’), than Balb/c mice.

In order to explore whether these behavioural (and cellular) differences between the two strains was due to pigmentation, we compared the degree of NIr penetration across the skin and fur in the different strains. In the Balb/c mice, we found that NIr penetration in the unshaved cases was 16% while in the shaved cases, it was 28%. In the C57BL/6 mice, NIr penetration was less, being 19% in the shaved cases and, quite remarkably, only 0.2% in the unshaved cases. Hence, these measurements indicated that the pigmented fur of the _C57BL_/6 mice absorbed almost all the NIr, hence limiting severely its penetration through to the brain.

## Discussion

We have two main findings. First, the MPTP-NIr group of Balb/c mice had greater locomotor activity and, as shown previously (Shaw et al. 2010), more surviving dopaminergic cells than the MPTP group. Second, these differences in cell survival and locomotor activity between the two groups were not as clear in C57BL/6 mice. Overall, our results indicated that Balb/c mice were a better model for exploring the neuroprotective effects of NIr after MPTP treatment than C57BL/6 mice.

### Comparison with previous studies

This study offers the first detailed description of changes in locomotor activity in MPTP-treated mice after NIr treatment. Whelan and colleagues [13] described briefly that NIr pre-treatment, but not post-treatment, improved locomotor activity in an acute MPTP mouse model (strain was not mentioned in that report). Our results in Balb/c mice confirms, at least in part, the results of that study.

There have been several previous reports on the behavioural and cellular changes in Balb/c and C57BL/6 mice after MPTP insult [17,18]. We confirm the findings of these reports in that there were fewer TH^+^ cells in the SNc of C57BL/6 mice than Balb/c mice (eg, saline controls) and that MPTP had a greater effect on locomotor activity in C57BL/6 than in Balb/c mice; further that Balb/c mice had some NIr-induced recovery of activity while C57BL/6 mice did not. Our results offered some differences to the previous studies, however. In particular, previous studies using non-stereological methods have reported a greater MPTP-induced cell loss in C57BL/6 compared to Balb/c mice [17,18]; our stereological analysis, by contrast, revealed a comparable loss in the two strains (~30%). The reason for these differences is not clear but they may reflect, for example, differences in our MPTP regimes (eg 50 mg/kg over 24 hrs vs. 60 mg/kg over 8 hrs) [17], methods of MPTP delivery (eg, intraperitoneal vs. intraventricular) [18] and methods of cell analysis (stereological vs. non-stereological) [17,18]. Finally, our control Balb/c mice had slightly better locomotor activity at baseline than the C57BL/6 mice, while Sedelis and colleagues [17] have reported the opposite. This discrepancy may reflect differences in the behavioural tests used and our measures of locomotor activity. For example, we measured velocity, high mobility and immobility using contrast changes, while the previous study recorded distance travelled with laser beam technology. Despite these differences in our studies, the key issue is that our MPTP regime was effective in generating TH^+^ cell loss and behavioural changes in the two strains, thereby allowing an assessment of neuroprotection by NIr treatment.

It should be noted that in this study, we did not undertake an analysis of the density of TH^+^ terminals in the striatum, nor of the locomotive activity of the mice after six days, the end of the experimental period. Previous studies have shown a complete recovery of TH^+^ terminal density in the striatum [18] and locomotive activity after six days in Balb/c mice using an acute model [19]; in C57BL/6 mice, although there are fewer TH^+^ terminals in the striatum of MPTP-treated animals compared to controls at this stage [18], the locomotive activity has been shown to return to control levels [19]. Hence, from these data, there would have been no point for us to explore these issues, mainly because any impact of NIr treatment - the central issue considered in the present study - would not have been elucidated.

### NIr treatment improved locomotor activity after MPTP insult in Balb/c mice

Our results showed that NIr treatment improved locomotor activity after MPTP insult in Balb/c mice, hence confirming the histological findings that there were more dopaminergic cells in MPTP-NIr than in MPTP groups [11,12]. The beneficial effect of NIr treatment was not immediate. It was only after the second MPTP injection (and subsequent NIr treatments; T3 and T4) that a clear difference in locomotor activity was recorded between the MPTP-NIr and MPTP groups. Before then (T1 and T2), no differences were evident between these two groups (with the MPTP effect being similar and immediate in both groups). Hence, it appears that it takes several doses of NIr treatment to elicit a beneficial outcome. The mitochondria of the dopaminergic cells, after the third and fourth NIr treatment, may have been stimulated further to increase ATP synthesis and reduce the production of reactive oxygen species [14,15], thereby being better prepared to protect against the second MPTP insult. It is noteworthy that Whelan and colleagues [13] reported improvement of locomotor activity in MPTP-treated mice after several NIr pre-treatments, but not after a single post-treatment. Indeed, previous studies reporting beneficial results in the majority of systems have used multiple NIr treatments of ~4 J/cm^2^[14,15]. There may well be a therapeutic window for NIr treatment and this may vary for different animals and systems [15].

### Strain differences in the effectiveness of NIr treatment after MPTP insult

Somewhat surprisingly, the beneficial effects of NIr treatment after MPTP insult were not as clear in the C57BL/6 mice. When compared to the Balb/c mice, the C57BL/6 mice had a smaller increase in dopaminergic cell number (20% vs 30%) and no clear improvement in locomotor activity in the MPTP-NIr compared to the MPTP group, at least over the later part of the survival period used in this study. Future studies may explore if there is a linear correlation between cell pathology and behavioural decline (and recovery) [28] in different strains of MPTP-treated mice after NIr treatment in the long-term; further, it would be of interest to examine if the finer details of motor disturbances in mice after MPTP treatment are improved after NIr treatment in the different mouse strains [29].

The reason for this strain difference was likely to be due to the pigmented fur of the C57BL/6 mice absorbing the majority of the NIr, preventing penetration into the brain. Our measurements indicated that in unshaved C57BL/6 mice, unlike in the shaved C57BL/6 and Balb/c (shaved and unshaved), there was very little NIr penetration (>1%). Melanin is certainly capable of absorbing the 670 nm wavelength [30] and that seemed sufficient to limit neuroprotection in the C57BL/6 mice. It is of course possible that, in addition to these penetration issues, the albino and pigmented strains have distinct cellular enzyme differences also, responsible for the different responses to NIr-induced metabolic (and therefore therapeutic) changes.

## Conclusions

In summary, although our results are in an animal model of the disease, a key point is that NIr appeared to have neuroprotective effects on structures deep in the brain. Our findings that NIr treatment reduced MPTP-induced degeneration among midbrain dopaminergic cells and improved locomotor activity in Balb/c mice, due to greater NIr penetration through skin and fur, form templates for future endeavour. It remains to be determined if NIr, when applied from an external device, is able to penetrate the thicker skull and meningeal layers, together with the greater mass of brain parenchyma to reach the SNc of humans.

## Abbreviations

CoQ10: Coenzyme Q10; ATP: Adenosine-5'-triphosphate; LED: Light emitting device; MPTP: 1-methyl-4-phenyl-1,2,3,6-tetrahydropyridine; NIr: Near-infrared light; PBS: Phosphate buffered saline; SNc: Substantia nigra pars compacta; SNr: Substantia nigra pars reticulata; TH: Tyrosine hydroxylase.

## Competing interest

There was no conflict of interest for any of the authors: CM,NT, DR, DJ, JS, ALB and JM are full-time members of staff at their respective institutions, while CP and NEM are undergraduate students.

## Authors’ contribution

All authors contributed to the analysis of the data and the writing of the manuscript. CM, NT, NEM, DR and JM contributed to the experimental work. All authors read and approved the final manuscript.
